# Efficacy of Chitosan, Pectin and Xanthan as Cold Gelling Agents in Emulsion Gels Stabilized with Legume Proteins to Be Used as Pork Backfat Replacers in Beef Burgers

**DOI:** 10.3390/gels9120970

**Published:** 2023-12-11

**Authors:** Nicoleta Cîrstea (Lazăr), Violeta Nour, Alexandru Radu Corbu, Georgiana Gabriela Codină

**Affiliations:** 1 Faculty of Food Science and Engineering, Dunărea de Jos University of Galati, Domnească Street 111, 800201 Galati, Romania; nl135@student.ugal.ro; 2 Department of Horticulture & Food Science, University of Craiova, 13 AI Cuza Street, 200585 Craiova, Romania; alexandru.corbu@edu.ucv.ro; 3 Faculty of Food Engineering, Stefan Cel Mare University of Suceava, 720229 Suceava, Romania; codina@fia.usv.ro

**Keywords:** emulsion gels, lipid composition, oxidative stability, pea protein isolate, soy protein isolate, technological properties, texture

## Abstract

This study aimed to develop stable emulsion gels enriched in polyunsaturated fatty acids, formulated with a mixture of olive (75%) and linseed (25%) oils, by incorporating two different stabilizers—pea and soy protein isolates—and three different cold gelling agents—chitosan, pectin and xanthan—to be used as pork backfat replacers in beef burgers. The color, pH, stability and textural properties of the emulsion gels were analyzed as affected by cold storage (4 °C, 7 days). Proximate composition, fatty acid content, technological and sensory properties were determined after burger processing. Meanwhile, color, pH, textural parameters and lipid oxidation were monitored in burgers at 0, 5 and 10 days of storage at 4 °C. A reduction of the fat content between 21.49% and 39.26% was achieved in the reformulated burgers as compared with the control, while the n-6/n-3 polyunsaturated fatty acid ratio decreased from 5.11 to 0.62. The highest moisture and fat retention were found in reformulated burgers made with xanthan, both with pea and soy proteins; however, their textural properties were negatively affected. The reformulated burgers made with chitosan were rated highest for sensory attributes and overall acceptability, not significantly different from the controls.

## 1. Introduction

The quality of meat and meat products and their role in human nutrition are currently the subject of numerous debates. Although the content of high-quality proteins, vitamins (e.g., B and D vitamins, retinol) and several bioavailable minerals (e.g., iron and zinc) in meat is undeniable [1], numerous epidemiological studies have shown that high consumption of meat products is associated with an increased risk of cardiovascular and other chronic diseases, largely related to the high-fat content of these products and their fatty acid profile, dominated by saturated fats [2,3]. Faced with the interest of consumers in limiting their intake of saturated fats and being attracted to healthier meat products, the meat industry is challenged to reformulate meat products by replacing added animal fats with healthier vegetable (olive, chia, linseed, walnut) or marine (fish, algae) oils in order to improve their fatty acid profile and reduce cholesterol [4,5]. However, the more saturated animal fats have positive impacts on the technological properties of meat products, such as emulsion stability and cooking yield, and sensory features, such as texture, mouthfeel and juiciness [6,7]. As a result, simply replacing animal fat with vegetable oils can have negative consequences on these properties [5,8]. To address these problems, the researchers are constantly concerned with developing new solid fat mimetics, consisting of oil-in-water emulsions stabilized in gel-like network structures combining aggregated emulsion droplets and cross-linked biopolymer molecules, able to capture the oil droplets and to mime the textural and technological properties of animal fats [9,10,11,12,13,14]. These composite gel systems made through the incorporation of a cold gelling agent in a protein-stabilized emulsion may achieve better textural features, higher water-holding capacity, and lower cooking loss [15].

The emulsion gels can be prepared by heat setting, acid- or enzyme-induced gelation or by adding cold gelling agents like polysaccharides, proteins or their combination [11,14,15,16]. Among the polysaccharides, carrageenan, alginate, chia flour, chitosan and inulin have been previously used as cold gelling agents to formulate emulsion gels, successfully used as a fat replacer in different meat products such as frankfurters [10,17,18,19], Bologna sausage [20,21], dry fermented sausages [11], burgers and pork patties [14,22].

Soy proteins are increasingly used as a stabilizer in emulsion gels since, apart from their nutritional value, availability, low price and health benefits, they can provide desired functional properties, including gelling, emulsifying, fat-absorbing and water-binding properties [23]. Likewise, the nutritional characteristics, low price, health benefits and functional properties (emulsifying, gelling and foaming) of pea proteins make them a promising food ingredient and a valuable alternative to soy proteins [23,24,25]. The hydrophobicity and molecular flexibility of soy and pea proteins make them good emulsifiers, allowing their rapid absorption at the interface to form a coherent macromolecular protective layer and confer interfacial stability. However, the pea protein isolate was demonstrated to have inferior gelling properties as compared with soy protein isolate [26]; therefore, the addition of a gelling agent would be helpful to improve its gelation ability. Polysaccharides, such as alginate, carrageenan, dextrin, inulin, konjac or chitosan, have been previously used in emulsion gels as cold gelling agents due to their hydrophilicity, high molecular weight and gelation capacity. These functional properties allow them to form a macromolecular barrier by increasing the viscosity of the aqueous phase and by retarding the coalescence between oil droplets. Chitosan is a biopolymer with a good gelation ability, widely studied to make films and coatings used to prolong the shelf life of fruits and vegetables [14]. Pectin is also a high-molecular-weight water-soluble polysaccharide with great potential for making water-soluble gels [27]. Xanthan is a polysaccharide largely used in the food industry owing to its water retention and texture improvement properties, mainly as an emulsion stabilizer and thickener and, secondarily, as a gelling agent [7]. Xanthan can stabilize emulsions, but in terms of gelation properties, it can form only weak gel structures and it performs as a stabilizing agent in emulsion systems only in combination with proteins [28]. To the best of our knowledge, there are only a few studies on the employment of chitosan, pectin or xanthan in the formulation of emulsion gels used as a fat replacer in meat products [7,14,21,29,30].

The high content of monounsaturated fatty acids (mostly oleic acid) and naturally occurring antioxidants makes olive oil a good candidate to be incorporated in the emulsion gels used as animal fat replacers in meat products [7,12]. Linseed oil could also be successfully added in emulsion gels to increase the content of n-3 polyunsaturated fatty acids (n-3 PUFAs) in meat products due to its high level of α-linolenic acid (~55%) [4,11].

The present study aimed to evaluate the effects of totally replacing the pork backfat with emulsion gels enriched in polyunsaturated fatty acids, manufactured using legume proteins such as soy and pea protein isolates as stabilizers, and polysaccharides such as chitosan, pectin and xanthan as cold gelling agents, on the nutritional, technological, textural and sensorial attributes of beef burgers.

## 2. Results and Discussion

### 2.1. Color, pH and Stability of Emulsion Gels

Stable, homogeneous emulsion gels with yellowish-cream color and solid-like properties were obtained and further used to substitute the pork backfat in burgers. The results of color parameters (L*—lightness, a*—redness and b*—yellowness), pH and total fluid release (TFR, %) of emulsion gels at 0 and 7 days of refrigerated storage (4 °C) are presented in Table 1.

The color of the emulsion gels is generally influenced by the protein used as a stabilizer and by the oil mixture incorporated in the gel. In turn, the emulsion gel color influences the color of the meat product. Just after processing, significantly (*p* < 0.05) higher L* values and lower a* values were observed in the gels stabilized with soy protein as compared with those made with pea protein. A loss of brightness (lower L* values) was observed in most samples during storage, while the a* and b* values generally remained at similar levels (*p* > 0.05).

The pH values were influenced mostly by the cold gelling agent. pH was significantly higher (*p* < 0.05) in the emulsion gels made with chitosan followed by those made with xanthan. Higher pH values generally determine a better water-holding capacity in meat products [31,32]. In addition, pH values impact the emulsifying capacity. Previous studies reported that formulations with higher pH values were those with better emulsion stability and attributed this behavior to the higher deviation from the isoelectric point of the meat and plant proteins, which affects the net charge of the protein. This contributes to a stronger and more cohesive protein interface on the fat particles and increases the protein interaction with water [25]. No significant (*p* < 0.05) pH variation was recorded in emulsion gels during 7 days of refrigerated storage.

Emulsion stability is an indicator of the ability of the emulsion system to retain water and fat after processing, and it is a useful technological property in cooked meat products. Since the emulsion gels were to be used to prepare the burgers, their thermal stability was measured and expressed as total fluid release (TFR, %). An improved emulsion stability could reduce water loss during cooking, thus influencing the stability and cooking yield of the meat product. Both proteins and polysaccharides influenced the stability of the emulsion gels. Previous studies demonstrated the great capacity of soy and pea protein to bind water and fat and their good emulsifying properties [33,34,35]. However, more stable emulsion gels were achieved from soy protein isolate as compared with those made with pea protein isolate, both immediately after processing and after 7 days of storage. Tang et al. [36] also reported that soy protein isolate had better emulsification stability than pea protein isolate. As regards the influence of the cold gelling agent, the emulsion gels made with xanthan showed the highest stability against centrifugation and thermal treatment, followed by those made with chitosan, while the stability of the emulsion gels made with pectin was much lower. TFR values between 0.11% and 2.43% were found for emulsion gels made with chitosan and xanthan. Similar values of TFR (%), ranging between 0.66 and 1.7%, have been previously reported for emulsion gels formulated using soy protein isolate, meat protein and microbial transglutaminase [37,38]. Also, high stability against centrifugation and thermal treatment, with no observable fluid leakage during 7 days of storage, was previously reported by Öztürk-Kerimoğlu [6] for a gel system comprising pea protein and agar-agar. Wang et al. [39] found also that the addition of chitosan to pork meat emulsions determined the enhancement of the emulsion stability due to the capacity of chitosan to form protein-polysaccharide complexes with meat proteins, which could improve water and fat retention during thermal treatment by facilitating absorption at the oil-water interface and by increasing the thickness of the interfacial layer and the steric barrier effects between oil droplets. Other studies have been carried out on the use of chitosan to improve emulsification in mayonnaise preparation [40].

### 2.2. Texture Analysis of Emulsion Gels

The values of the texture parameters for the emulsions studied are presented in Table 2. The results showed that both the protein and the cold gelling agent influenced the texture parameters of the emulsions. After processing, the emulsions made from soy protein were harder than those prepared with pea protein, despite the higher protein content of the latter. Žugčić et al. [41] found that beef patties containing soy protein had higher hardness, gumminess, and chewiness than those containing pea proteins. The hardest and the most gummy, chewy and adhesive emulsions resulted from the incorporation of chitosan as a cold-gelling agent, while the use of pectin determined the formation of the least adhesive emulsions. Cohesiveness and springiness were not significantly (*p* < 0.05) affected by either the protein or the cold gelling agent used in the emulsion formulation. Wang et al. [39] also reported that the incorporation of chitosan combined with chickpea protein isolates in pork meat emulsions significantly increased hardness and chewiness (*p <* 0.05) but did not influence the springiness and cohesiveness (*p >* 0.05) of phosphate-free pork meat emulsions. Other studies also demonstrated that chitosan can induce a firm texture in meat products by improving the interactions with the negative charges of proteins and enhancing the gelation ability of meat emulsions [42]. After storage, the hardness decreased while adhesiveness slightly increased in all samples.

### 2.3. Proximate Composition and Energy Values of Burgers

The results for the proximate composition of control and reformulated burgers are presented in Table 3. The burgers obtained through reformulation (PPI-CH, PPI-P and PPI-X—burgers reformulated with EG-PPI-CH, EG-PPI-P and EG-PPI-X, respectively; SPI-CH, SPI-P and SPI-X—burgers reformulated with EG-SPI-CH, EG-SPI-P and EG-SPI-X, respectively) were low-fat burgers because their fat content, which was in the range of 10.46% to 13.52%, represents a reduction between 21.49% and 39.26% compared to the fat content of control burgers (Control).

The lowest reductions in the fat content were registered in burgers made with emulsion gels using pectin as a cold gelling agent (21.49% and 23.01% for PPI-P and SPI-P, respectively) while the highest was registered in burgers reformulated with emulsion gels stabilized with chitosan (39.26% and 34.56% for PPI-CH and SPI-CH, respectively). The moisture content was significantly (*p* < 0.05) higher in all reformulated burgers as compared with the controls. These results could be due to the high water-holding capacity of both proteins and polysaccharides. The highest increases in moisture content were found in the burgers using xanthan as a cold gelling agent in the emulsion gels substituting the pork backfat in the burger’s formula. Other previous studies also reported the extraordinary capacity of xanthan to improve the water-holding ability of low-fat emulsions [43,44].

Xanthan is a hydrocolloid gelling gum that, similar to other polysaccharides, interacts with proteins, thus affecting the gel-forming ability and water-holding capacity of proteins and impacting the structure and stability of foods [45]. Unlike xanthan, which dissolves in cold water forming viscous solutions with a weak gel character, pectin is a branched macromolecule of high molecular weight that has swelling properties but does not dissolve in water [46,47]. Chitosan, which is also water-insoluble but soluble in weak organic acid solutions, has been demonstrated to possess properties for use in water and fat retention, emulsification and gelation [40,48]. Amaral et al. [42] reported that the incorporation of 2% (*w*/*w*) chitosan in goat sausages showed the ability to bind water and fat, thus improving the characteristics associated with cooking as compared with control samples. Our results showed that chitosan was significantly (*p* < 0.05) more effective in retaining water than pectin, both in combination with pea and soy protein isolate. These results were in good agreement with those reported by Han and Bertram [49], showing that chitosan promoted more cross-links between meat matrix components than several common polysaccharides, including pectin, in comminuted meat products.

The percentage protein content raised in all reformulated burgers, except those incorporating xanthan, but this could be due to the higher moisture content of these samples. Higher protein contents were found in samples reformulated with emulsions based on pea protein isolate as compared with those based on soy protein isolate, which was to be expected considering that an incorporation of 16% pea protein isolate was required to obtain the desired consistency for the emulsion gels, as compared with an incorporation level of 10% for the soy protein isolate.

As a result of reducing the fat content, the energy value of the burgers also decreased through reformulation. The highest decreases in the energy values were found in the samples incorporating xanthan (22.70% and 23.07% for PPI-X and SPI-X, respectively), while the lowest reductions were found in the burgers incorporating pectin (9.60% and 11.91% for PPI-P and SPI-P, respectively).

Moisture contents were generally higher in the reformulated samples incorporating soy protein isolate compared to those with pea protein isolate having the same cold gelling agent, probably as a result of the higher water-holding capacity of soy protein as compared to pea protein. Many previous studies demonstrated the poor water-binding properties of the pea protein relative to soy and attributed the differences to the protein’s structure, as soy proteins, having more hydrophilic groups near the surface, retain more water [50,51,52].

### 2.4. Fatty Acid Profile

The fatty acid profiles of the control and reformulated burgers are shown in Table 4. The average fatty acids content was used to calculate total saturated (SFA), total monounsaturated (MUFA), total n-3 polyunsaturated (PUFAn-3), total n-6 polyunsaturated (PUFAn-6) and total polyunsaturated (PUFA) fatty acids. The PUFA/SFA and n-6/n-3 ratios, the atherogenic (AI) and thrombogenic (TI) indexes and the ratio of hypocholesterolemic and hypercholesterolemic fatty acids (h/H) were calculated as indicators of dietary fat quality. The substitution of the pork backfat with emulsion gels made with a mixture of olive (75%) and linseed (25%) oils in burgers significantly influenced the fatty acid profile. The major fatty acid in control samples was oleic (C18:1n-9, 35.18 g/100 g), followed by palmitic (C16:0, 27.95 g/100 g) and stearic (C18:0, 13.19 g/100 g) acids. After reformulation, the oleic acid content increased in the range of 33.17–35.36%, nervonic acid was also detected at levels of 1.75–1.92 g/100 g, while palmitic and stearic acid contents decreased by 57.38–58.74% and 63.60–64.82%, respectively. No significant differences were found between the reformulated burgers for any of these fatty acid contents. The major polyunsaturated fatty acid in the control burgers was linoleic (C18:2n-6, 5.17 g/100 g), followed by eicosadienoic (C20:2n-6, 1.75 g/100 g) and octadecatetraenoic (C18:4n-3, 1.18 g/100 g) acids. The total pork backfat replacement with emulsion gels produced a significant increase (*p* < 0.05) in linoleic acid (C18:2n-6) (by around two times) and arachidonic acid (C20:4n-6) (from 0.05 to around 0.37 g/100 g) together with a major increase in alpha-linolenic acid (C18:3n-3, from 0.13 g/100 g in control to 10.16–10.50 g/100 g in reformulated burgers). In addition, the eicosadienoic and octadecatetraenoic acid contents significantly (*p* < 0.05) decreased as a result of reformulation. The results for individual fatty acids content are in agreement with previous studies in which part or all of the pork fat in the meat product was replaced with olive oil [7,53,54] or with emulsions containing olive and linseed oils [55].

No significant differences were found between the reformulated samples concerning the SFA, MUFA, PUFA, PUFAn-6 and PUFAn-3 contents, which was to be expected since all the emulsifying gels formulated in the present study had the same oil mixture content.

The fatty acid profile of the control burgers was dominated by SFAs, followed by MUFAs. The SFA content decreased by around 63.5% while MUFA and PUFA content increased 1.3 and 3.4 times, respectively, in reformulated burgers as compared with the controls. These changes in the fatty acid profile as a result of reformulation may be assigned to the richness of olive oil in oleic acid and of the linseed oil in polyunsaturated fatty acids, mainly alpha-linolenic acid [56]. Of the PUFAs, PUFAn-6 content increased on average 1.57 times while PUFAn-3 achieved the highest multiplication ratio (12.86), mainly as a result of the increase in the α-linolenic acid content (C18:3n-3) from 0.13 to around 17.40 g/100 g. As a consequence, the n-6/n-3 ratio significantly dropped from 5.11 to around 0.62 (by more than 8 times) in the reformulated burgers as compared with the control, while the PUFA/SFA ratio increased from 0.17 to 1.56. Delgado-Pando et al. [57] also reported a reduction in the n-6/n-3 ratio from 9.27 to 0.47 and an increase in the PUFA/SFA ratio from 0.27 to 1.7 after replacing pork backfat with an emulsion gel based on olive, linseed and fish oils in low-fat frankfurters. Meanwhile, Franco et al. [4] found a decrease in the n-6/n-3 ratio from 14.92 to 1.61 and an increase in the PUFA/SFA ratio from 0.47 to 0.78 as a result of substituting 50% of pork backfat with a linseed oleogel. Increasing the intake of PUFAn-3 is important, as the eicosanoids synthesized from PUFAn-3 appear to have anti-inflammatory and anticancer effects, while those derived from the n-6 series of PUFAs promote inflammation and other pathologies [58]. Currently, it is known that a low n-6/n-3 ratio has favorable effects on lipid metabolism and vascular endothelial function and makes contributions to the prevention and treatment of cardiovascular and cerebrovascular diseases [59].

The improvement of burger healthiness was also proved by the decrease in the atherogenic index from 0.80 to 0.16–0.17 and the thrombogenic index from 1.34 to 0.19–0.20, as well as by the increase in the h/H ratio from 1.33 to 6.09–6.35 as a result of the reformulations. These results agree with those previously reported for reformulated meat products made by substituting animal fat with vegetable oils [5,18,60]. The reformulated burgers could be claimed as “high unsaturated fat” according to Regulation (EC) No 1924/2006 and Commission Regulation No 432/2012, since at least 70% of the fatty acids (80.07–80.82%) derive from unsaturated fat.

### 2.5. Texture Analysis of Burgers

The texture parameters of burgers showed high variation between formulations (Table 5). Reformulation affected all of the textural properties of burgers (except springiness). The observed differences in textural parameters could be attributed to the proteins as well as to the cold gelling agents used in the emulsion gel formulation.

The replacement of pork backfat with emulsion gels made with chitosan or pectin significantly (*p* < 0.05) increased hardness, shear force, resilience, gumminess and chewiness as compared with the controls. Cohesiveness increased in all samples as a result of the reformulation, and this may be attributed to both the proteins and polysaccharides used as a cold gelling agent. Previous studies also reported that the incorporation of soy proteins increased the cohesiveness of emulsified meat products [61].

After processing, the adhesiveness of all reformulated burgers was higher as compared with the control samples, but the highest adhesiveness was found in burgers made with emulsion gels incorporating xanthan, with both soy and pea proteins. The burger formulations made with chitosan (PPI-CH and SPI-CH) registered the highest hardness, shear force and cohesiveness, but also the highest gumminess, while those made with xanthan as a cold-gelling agent, with both pea and soy proteins, registered the lowest hardness, shear force and chewiness, significantly (*p* < 0.05) lower than those of the control burgers. These results could be related to the higher hardness of the emulsion gels made with chitosan (as previously presented in Table 2), but also to the higher water loss found in the burgers reformulated with emulsion gels based on chitosan, as it is well-known that water influences texture by acting as a plasticizer, with meat products with higher water loss presenting a higher hardness [25]. Based on the same principle, the lowest hardness values found in the burgers reformulated with emulsion gels based on xanthan (PPI-X and SPI-X) could be attributed to their highest moisture retention. Majzoobi et al. [62] also reported that the addition of xanthan gum reduced the hardness and chewiness of the meat-free sausages, particularly at concentrations higher than 0.6%. This occurs when the competition of xanthan and soy proteins for water absorption leads to incomplete development of soy protein, and hence, to the texture weakening of the emulsion.

As a protein ingredient, the gel-forming and water-retention abilities of soy proteins are well recognized. Previously, it was demonstrated that soy protein isolate gels had higher elasticity and hardness and stronger rheological properties than pea protein isolate gels under the same gelation conditions [50]. However, the burgers reformulated with emulsion gels based on pea protein were harder and more cohesive than those with soy protein, probably due to the higher protein content of the former.

### 2.6. Color and pH of Burgers

Table 6 shows the results of pH and color parameters of control and reformulated burgers at 0, 5 and 10 days of refrigerated storage (4 °C). The lightness (L* values) significantly (*p* < 0.05) decreased while the yellow coordinate (b*) significantly (*p* < 0.05) increased in burgers reformulated by replacing pork backfat with emulsions based on chitosan or pectin, both with pea and soy protein isolates, while in those incorporating xanthan, no significant differences were found in these color parameters as compared with the controls. The lower lightness observed after cooking in reformulated meat products as compared with the controls has been previously observed, and it was attributed to the browning of soybean or pea proteins during the Maillard reaction [63]. The Maillard reaction is also assumed to be the reason for the increase in yellowness (b* values) in the reformulated samples as compared to the control. Revilla et al. [7] reported a progressive decrease in L* values and an increase in b* values as a result of the substitution of meat with texturized pea protein and attributed these variations to the intense yellow color of texturized pea protein. Wang et al. [39] found also that b* values increased significantly after adding chickpea protein isolate and chitosan (*p <* 0.05) to phosphate-free pork meat emulsions. Redness (a* values) significantly increased through reformulation only in burgers incorporating chitosan, whereas in the other formulations, a* values did not show significant (*p* < 0.05) differences.

The results indicate a general trend of decreasing a* values and increasing b* values during storage in all samples. No significant differences were found (*p* < 0.05) between the color parameters of the burgers made with pea and soy protein isolate and incorporating the same cold gelling agent.

The pH values were significantly higher (*p* < 0.05) only in the burgers reformulated by substituting the pork backfat with emulsion gels made with chitosan (Table 6). Regarding the evolution during storage, the pH tended to increase, but it did not change significantly (*p* < 0.05) during 10 days of refrigerated storage.

### 2.7. Technological Properties

The technological properties of control and reformulated burgers are presented in Table 7. The results showed that cooking loss was significantly (*p* < 0.05) lower in reformulated burgers made by replacing pork backfat with emulsion gels made with chitosan or xanthan as compared with the controls. Other previous studies also reported a decrease in cooking loss in beef burgers in which animal fat was replaced by emulsion gels based on vegetable oils [60,64,65]. The reformulated burgers made with pectin showed higher cooking loss than the controls, although the differences did not reach statistical significance. The higher cooking loss found in burgers reformulated with pectin (PPI-P and SPI-P) was to be expected considering the higher total fluid release of the emulsion gels made using pectin as a cold gelling agent, as presented in Table 1.

Shrinkage is generally determined by protein denaturation, moisture and fat release during cooking [66]. As previously found in other studies, the results for shrinkage were in good agreement with those found for cooking loss [67].

Moisture and fat retention in meat products are important technological properties since retained moisture and fat affect sensory quality and acceptability. Moisture retention was significantly higher (*p* < 0.05) in all reformulated burgers as compared with the controls. This might be attributed to the water and oil holding capacity of both polysaccharides and proteins incorporated in the emulsion gels, as well as their interaction with the meat proteins.

The highest moisture retention was found in the reformulated burgers made with xanthan, with both soy and pea protein isolate. The lowest fat retention was found in the control samples due to the higher fat content and the melting of pork backfat globules. The burgers reformulated by replacing pork backfat with emulsion gels made with pectin showed the highest cooking loss and the lowest moisture retention; however, the highest fat retention was found in these formulations. The technological properties are comparable for the two proteins at the incorporation levels used in the emulsion gels (16% pea protein isolate and 10% soy protein isolate). However, Tang et al. [36] reported better water and oil holding capacities of the soy protein isolate as compared with the pea protein isolate. They also reported a least gelation concentration (LGC) of 10% for the soy protein isolate and 16% for the pea protein isolate, in good agreement with the findings of our preliminary studies that led to the protein concentrations used in the emulsion gel formulations.

### 2.8. Lipid Oxidation

**Figure 1:**
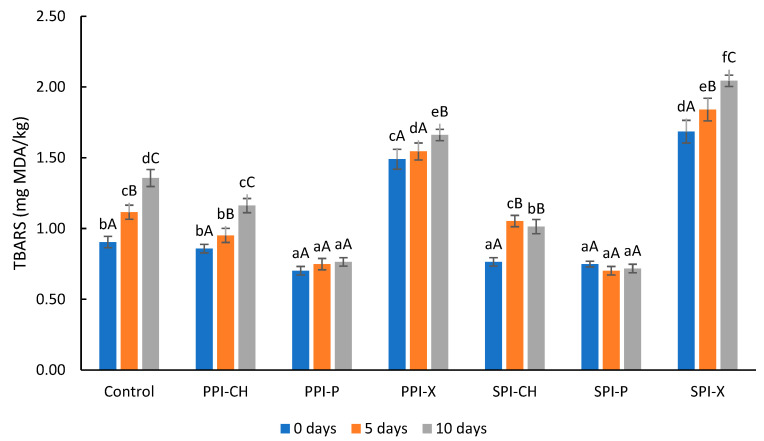
TBARS values (mg MDA/kg) in control and reformulated burgers at 0, 5 and 10 days of storage at 4 °C. Different lowercase letters indicate significant differences between burger formulations (*p* < 0.05) for the same storage period, while different uppercase letters are indicative of significant differences between sampling times for the same burger formulation (*p* < 0.05); Control—control burgers; PPI-CH, PPI-P and PPI-X—burgers reformulated with EG-PPI-CH, EG-PPI-P and EG-PPI-X, respectively; SPI-CH, SPI-P and SPI-X—burgers reformulated with EG-SPI-CH, EG-SPI-P and EG-SPI-X, respectively.

By reformulation, the PUFA content of the burgers increased together with their unsaturation degree and with their susceptibility to oxidation [5,68]. Monitoring lipid oxidation during the shelf life of burgers is important, as oxidation is responsible for the degradation processes and the formation of off-flavors and toxic substances. The results of the concentrations of thiobarbituric acid-reactive substances (TBARS) values in control and reformulated burgers at 0, 5 and 10 days of refrigerated storage (4 °C) are presented in Figure 1.

Just after processing, TBARS values were lower in burgers reformulated by replacing pork backfat with emulsion gels made with pectin or chitosan. Other authors reported the same trend in their studies when animal fat was replaced by an emulsion gel incorporating vegetable oils in various meat products. These findings have been attributed to the combined effects of (a) the lower fat content in the reformulated products, (b) the presence of natural antioxidants in the oil mixture used to formulate the emulsion gels, (c) the antioxidant capacity of some gelling agents as well as (d) the protection provided by the emulsion gel by oil immobilization [22,69]. In our study, the reformulated burgers also had lower fat content, and the extra virgin olive oil incorporated in the emulsion gels contributed to the antioxidant protection through its high content of tocopherols and phenolic compounds [70].

However, TBARS values were significantly higher in burgers reformulated with emulsion gels containing xanthan as a cold gelling agent as compared with the other samples, which proves that the gelling agent still plays an important role in the antioxidant protection of the meat product. The failure to provide good antioxidant protection in these samples could be attributed to the inability of the gel based on xanthan to function as a good oxygen barrier in conjunction with the higher water retention and the weaker and porous structure of these burgers. Li et al. [71] also reported that a lower cooking loss and higher moisture content were significantly correlated (*p* < 0.05) with protein oxidation in roasted beef patties.

Lipid oxidation increased during storage both in control and reformulated burgers made with chitosan and xanthan, while in the samples made with pectin, the increment of malonaldehyde (MDA) content was not statistically significant. After processing, all the burgers showed oxidation values within acceptable limits, but after 10 days of storage, the control and the reformulated burgers made with xanthan recorded TBARS values above the threshold of 1.0 mg MDA/kg, at which consumers may detect rancidity [68].

### 2.9. Sensory Analysis

Sensory attributes are one of the limiting factors for fat-replacing strategies due to the importance of fat in the sensory palatability of meat products [66]. The effect of reformulations on the sensory properties of burgers is shown in Table 8. The scores awarded by the panelists for taste and flavor were higher in controls as compared with the reformulated burgers; however, the differences were not statistically significant (*p* < 0.05). In terms of appearance, the lowest scores were achieved by the reformulated burgers made with xanthan, mainly as a result of the ragged appearance of these burgers. Serdaroğlu et al. [66] also found that replacing beef fat with gelled emulsion prepared with olive oil showed a negative impact on the sensory characteristics of chicken patties. The reformulated burgers made with chitosan or pectin were darker as compared with the controls, as revealed by instrumental color analysis (Table 6). However, their appearance scores were not significantly different from the controls. Significant differences (*p* < 0.05) were observed in texture, mainly because the texture of the reformulated burgers made with xanthan was seriously affected. Although these samples were extremely juicy and tender due to the greater moisture retention, they were extremely crumbly, a feature that is not desirable in burgers. The textural defects of these samples affected their general acceptability, which was significantly lower than that of the other samples.

The reformulated burgers made with chitosan were rated highest for overall acceptability, not significantly different from the controls. These findings are consistent with previous studies that found no significant differences in overall acceptability when pork backfat was replaced by emulsion gels made with vegetable oils and cold gelling agents [18,37]. The protein used in the present study did not significantly influence the sensory properties of the burgers.

## 3. Conclusions

The addition of soy and pea protein isolate as stabilizers in emulsion gels at concentrations of 10% and 16%, respectively, in combination with chitosan, pectin or xanthan as a cold gelling agent, allowed the obtainment of emulsion gels with appropriate physical-chemical and textural properties, able to replace pork backfat in beef burgers.

The reformulation of burgers, achieved by the total replacement of pork backfat with the emulsion gels formulated in this study, significantly improved their lipid profile and healthiness. The replacement of pork backfat with the emulsion gels made with chitosan or pectin significantly increased the hardness and oxidative stability of the burgers as compared with the controls. The highest moisture and fat retention were found in the reformulated burgers made with xanthan, with both pea and soy proteins. However, the textural properties and oxidative stability of these burgers were negatively affected. The replacement of pork backfat with emulsion gels stabilized with chitosan was the best formulation, improving the nutritional value, technological parameters and oxidative stability of beef burgers without negatively impacting their sensory properties and general acceptability.

## 4. Materials and Methods

### 4.1. Materials

Post-rigor (48 h postmortem) round beef lean meat and pork backfat were both purchased from a local meat market in Craiova (Romania) and kept refrigerated at 4 °C until use. The pea protein isolate (PPI) (Bio Pea Protein, protein content 80%) was provided by GymBeam (Košice, Slovacia), while the soy protein isolate (SPI) (Supro Ex 37, protein content 91.8%) was from Solae Belgium N.V. (Ieper, Belgium).

The extra virgin olive oil was from the Monini brand (Spoleto, Italy), while linseed oil was from Herbavit (Oradea, Romania). According to the information provided by the suppliers, the linseed oil contained 8% SFA, 22% MUFA and 70% PUFA, while for the extra virgin olive oil a lipid composition of 15.2% SFA, 75% MUFA and 9.8% PUFA was provided. Chitosan (CH) from BiOrigins (Fordingbridge, UK), xanthan gum (X) from GymBeam (Košice, Slovacia) and pectin (P) from Sosa Ingredients (Moià, Spain) were the polysaccharides used as cold gelling agents in the emulsion gel formulations. Microbial transglutaminase, specifically Activa WM, with a standard enzyme activity of approximately 100 U/g, was acquired from Ajinomoto Europe Sales GmbH (Hamburg, Germany). Malondialdehyde (>96%), trichloroacetic acid (>99%) and thiobarbituric acid (>98%) were purchased from Sigma-Aldrich (St. Louis, MO, USA). All other chemicals were of analytical grade and were supplied by Merck (Darmstadt, Germany).

### 4.2. Preparation of Emulsion Gels

Six emulsion gel formulations were elaborated based on preliminary testing, as shown in Table 9, to be used in burgers as pork backfat replacers.

All six emulsion gels included a fixed content of oil (40%) consisting of a mixture of 75% extra virgin olive oil and 25% cold-pressed linseed oil. The emulsion gels were prepared one day before burger processing according to the method described by Cîrstea et al. [14]. The emulsion gels were made using a BOSCH VitaBoost MMBH6P6 blender (Bosch, Stuttgart, Germany) at room temperature. The protein isolate (pea or soy) and microbial transglutaminase were homogenized at high speed with water (30 s, approx. 5600 rpm), then the cold gelling agent (chitosan, pectin, or xanthan) was added and further homogenized for another 15 s. Finally, the oil mixture previously obtained was gradually incorporated and homogenized in the same blender for a further 3 min to obtain a uniform emulsion. Each emulsion gel was prepared in three independent batches, and each batch (500 g) was placed in a plastic container and stored under refrigeration (4 °C) until use. The color, pH, stability determinations and textural analysis were performed after 24 h of refrigerated storage.

### 4.3. Preparation of Burgers

Seven different burger formulations of about 2 kg each were prepared. Three batches of each formulation were performed on different days. The control formulation, without replacement of pork backfat, was prepared as reference (Control), while the other six formulations were made by the total replacement of pork backfat with the corresponding emulsion gels previously developed, as follows: PPI-CH, PPI-P, PPI-X, SPI-CH, SPI-P, SPI-X by replacing pork backfat with EG-PPI-CH, EG-PPI-P, EG-PPI-X, EG-SPI-CH, EG-SPI-P, EG-SPI-X emulsion gels, respectively.

The burgers were made based on lean beef meat after cutting it into small chunks and mincing it in a meat grinder, specifically the BOSCH ProPower MFW68660 (Bosch, Stuttgart, Germany) using a 30-mm sized plate grinder. The pork backfat for the control burgers was also cut and minced using the same plate. Firstly, minced beef (78.5%) was mixed with salt (1.5%) to extract myofibrillar proteins. Then, minced pork backfat or emulsion gels (20%) were added, and the mixture was kneaded by hand until complete homogenization. The paste was divided into portions of 72.5 g and the burgers were formed by compacting the paste portions in a circular mold (70 mm diameter and 18 mm depth). Twenty-eight burgers were made for each of the seven experimental variants. After forming, the burgers were placed in a pre-heated hot air electric oven (Beko, BIM24300GPS, Istanbul, Turkey) at 180 ± 5 °C and allowed to cook for 40 min to attain the core temperature of about 73 ± 1 °C measured by a digital puncture thermometer inserted in the center of the burger. After cooking, the burgers were left to cool to room temperature, aerobically packaged and stored at a cold temperature (4 ± 1 °C) for 10 days.

The proximate and fatty acid compositions were determined after manufacturing and cooling the burgers (day 0). Determinations of color, pH and TBARS were carried out on day 0 and after 5 and 10 days of refrigerated storage. The sensory evaluation of the cooked burgers was performed just after manufacturing the burgers.

### 4.4. Color, pH and Stability of Emulsion Gels

Emulsion gel stability was measured on day 0 and day 7 and expressed as the total fluid release (TFR, %) according to Jiménez-Colmenero et al. [37], with slight modifications. About 25 g of emulsion samples were placed into pre-weight centrifuge tubes, hermetically sealed, heated in a water bath for 30 min at 70 °C and then centrifuged for 15 min at 2500× *g* using a Hermle Z300 centrifuge (Hermle Labortechnik, Wehingen, Germany). Afterward, the tubes were opened and left standing upside down for 50 min, to release the exudate onto a plate. The remaining emulsion was weighed, and the total fluid release was determined by the difference, then expressed as a percent of the initial emulsion weight. Three determinations were carried out for each formulation per batch.

The color of emulsion gels was measured on days 0 and 7 of storage using a PCECSM1 colorimeter (PCE Instruments, Southampton, UK) with spectral reflectance operating in the CIELab system to record L* (lightness), a* (redness) and b* (yellowness). Six determinations were carried out on the surface of three samples from each formulation and batch.

The pH of emulsion gels was determined in triplicate on day 0 and day 7 using a Hanna pH meter HI255 (Hanna Instruments, Padova, Italy). The analysis was carried out at room temperature on the emulsion gel homogenates, made by mixing them with water in a ratio of 1:10 (*w*/*v*).

### 4.5. Proximate Analysis

Moisture, fat, protein and ash content were determined in burgers according to the AOAC methods [72]. Moisture was determined based on the drying method (AOAC 950.46) using a Memmert ULM500 drying oven (Uden, The Netherlands) and the ash content based on the dry ashing method (AOAC 920.153) in a Caloris CL 1206 oven (Romania). An automatic Kjeldahl nitrogen analyzer (UDK 149 Velp Scientific, Milan, Italy) was used to determine the crude protein content based on the Kjeldahl method (AOAC 992.15), while a Soxhlet automatic extraction system (SER 148/3, Velp Scientific, Usmate, Italy) was employed to determine the fat content according to the Soxhlet method (AOAC 985.15). Proximate composition analyses were performed on raw and cooked burgers in two repetitions per batch of each formulation. The energy value was calculated based on 9.1 kcal/g for fat and 4.1 kcal/g for protein.

### 4.6. Fatty Acid Profile and Nutritional Indices

The fatty acid composition of the burgers was analyzed in triplicate in the lipid extracts by fatty acid methyl esters (FAMEs) gas chromatography, using a Perkin-Elmer Clarus 500 gas chromatograph (Shelton, MA, USA). The fatty acids were transesterified for 4 h with sulfuric acid in methanol (3% solution) at 80 °C to be converted to their methyl esters. The separation of FAMEs was carried out on a DB-23 GC capillary column (60 m × 0.25 mm id × 0.25 µm film thickness) from Agilent J&W GC Columns (Santa Clara, CA, USA). The temperature was programmed to increase at a rate of 5 °C/min from 180 °C to 220 °C. The carrier gas was hydrogen at 35 cm/s and 180 °C and the split ratio was 1:100. The injector and detector temperatures were 250 °C and 260 °C, respectively. FAMEs were detected by retention time with a flame ionization detector and identified by comparison with individual standard pure compounds from Sigma-Aldrich Chemical Co. (St. Louis, MO, USA). The fatty acid contents were calculated based on chromatogram peak areas and were expressed as g per 100 g total fatty acids. All analyses were performed in triplicate. The atherogenic (AI) and thrombogenic (TI) indexes were calculated according to Ulbricht and Southgate [73], and the ratio of hypocholesterolemic and hypercholesterolemic fatty acids (h/H) was calculated according to Santos-Silva et al. [74].

AI = (C12:0 + 4 × C14:0 + C16:0)/(Σ MUFA + Σ PUFA)

TI = (C14:0 + C16:0 + C18:0)/(0.5 × Σ MUFA + 0.5 × Σ PUFA n-6 + 3 × Σ PUFA n-3 + PUFA n-3/PUFA n-6)

h/H = (C18:1 n-9 + C18:2 n-6 + C20:4 n-6 + C18:3 n-3 + C20:5 n-3 + C22:5 n-3 +C22:n-3)/(C14:0 + C16:0)

### 4.7. Technological Properties

After cooling for 1 h at room temperature, the cooked burgers were weighed, and their diameter was measured with a vernier caliper. The total cooking loss (water + fat) was determined by the weight difference between fresh and cooked burgers and expressed as a percentage of the initial weight. Shrinkage was determined by the diameter difference between raw and cooked burgers and expressed as a percentage of the initial diameter [64]. The moisture retention and the fat retention were determined as shown below [75]. Ten samples were weighed and measured from each formulation and batch.

Cooking loss (%) = [(raw burger weight − cooked burger weight)/raw burger weight] × 100

Shrinkage (%) = [(raw burger diameter − cooked burger diameter)/raw burger diameter] × 100

Moisture retention (%) = [(100 − cooking loss (%)) × moisture in cooked burger]/100

Fat retention (%) = [(cooked burger weight × fat in cooked burger)/(raw burger weight × fat in raw burger)] × 100

### 4.8. Color Measurement

The color of burgers was measured on days 0, 5 and 10 of storage using a PCECSM1 colorimeter (PCE Instruments, Southampton, UK) operating in the CIELab system to record the color coordinates lightness (L*), redness (a*) and yellowness (b*). Six determinations were performed on the surface of the homogenized ground mixture of the three samples from each formulation and batch.

### 4.9. pH Measurement

The pH of burgers was measured in triplicate on days 0, 5 and 10 using a Hanna pH meter HI255 (Hanna Instruments, Padova, Italy). The analysis was carried out at room temperature on the burger homogenates made in distilled water at a ratio of 1:10 (*w*/*v*) by using a vertical blender (Braun MQ5137BK, 750W).

### 4.10. Textural Analysis

The textural characteristics of emulsion gels and cooked burgers were analyzed using a TVT-6700 texture analyzer from Perten Instruments (Hagersten, Sweden). For the burger samples, the behavior in both compression and cutting was evaluated. For the compression test, a texturometer equipped with a 10-kg load cell was used. Sample slices cut from the center of the burger, each 10 mm in height, were subjected to two compression cycles up to 50% of their initial height using a 20 mm-diameter cylindrical probe. A trigger force of 20 g was used at a speed of 1.5 mm/s, with a recovery time of 10 s between compressions. Six determinations were made for each formulation and batch. The following parameters were recorded: firmness (N), adhesiveness (J), resilience (adm.), springiness (adm.), cohesiveness (adm.), gumminess (N) and chewiness (N). For the cutting behavior, the Warner-Bratzler shear test was performed to measure the shear force (N). The texturometer device was equipped with a knife blade of 117 mm height with a probe holder. The determinations were performed in the center of each sample. The starting distance from the sample was 5 mm, the test speed was 1.5 mm/s and the trigger force was 40 g. For the emulsion gel samples, a double compression rate was applied to samples of 8 g weight, at 50% height, with a speed of 5.0 mm/s and a trigger force of 10 g. The recovery period between compressions was 120 s. All the determinations were made in triplicate on day 0 and day 7 for each sample.

### 4.11. Lipid Oxidation

The oxidative stability of burgers was assessed by monitoring the concentrations of thiobarbituric acid-reactive substances (TBARS) on day 0 and after 5 and 10 days of refrigerated storage according to the method of Witte et al. [76], with minor changes. Shortly, burger samples (5 g) were homogenized with 12.5 mL of 20% trichloroacetic acid, then diluted to 25 ml with cold distilled water. The resulting homogenate was filtered (Whatman No. 1) and the supernatant (5 mL) was mixed with 20 mM 2-thiobarbituric acid (5 mL). The absorbance was read at 532 nm on a Varian Cary 50 UV spectrophotometer (Varian Co., Palo Alto, CA, USA) after heating the mixture at 100 °C for 35 min and cooling it to room temperature. The analysis was performed on cooked burgers of each batch and formulation, with three replications per sample. A calibration curve made with 1,1,3,3-tetramethoxypropane was constructed, and results were expressed as mg malonaldehyde (MDA)/kg.

### 4.12. Sensory Analysis

Control and reformulated burgers were evaluated immediately after processing by a panel of twelve members comprising staff and master students from the Department of Food Science of the University of Craiova (Craiova, Romania). The sensory attributes were evaluated on a 9-point hedonic scale which ranged from “1-dislike extremely” to “9-like extremely” for the following attributes: appearance, taste, flavor, texture, and overall acceptability. Coded samples were offered to the panelists in a random order at a serving temperature of around 50 °C. Water and bread were served to the panelists to rinse the mouth between samples. A session was conducted for each batch, triplicate evaluations were made for each formulation and the average score was calculated for each attribute.

### 4.13. Statistical Analysis

The statistical analysis of the results was accomplished using Statgraphics Centurion XVI software (StatPoint Technologies, Warrenton, VA, USA). The statistical significance of the effect of emulsion gel or burger formulation was assessed by applying one-way analysis of variance (ANOVA) followed by the Fisher‘s least significant difference (LSD) test at a 95.0% confidence level. Furthermore, a two-way ANOVA followed by the LSD test (*p* < 0.05) was run to investigate the effect of formulation and storage time on pH, color and textural parameters, and TBARS values.

## Figures and Tables

**Table 1:** Color parameters (L*—lightness, a*—redness and b*—yellowness), pH and total fluid release of emulsion gels at 0 and 7 days of storage at 4 °C *.

Storage Time (Days)	EG-PPI-CH	EG-PPI-P	EG-PPI-X	EG-SPI-CH	EG-SPI-P	EG-SPI-X
L*						
0	65.17 ± 2.32 ^aB^	71.14 ± 2.19 ^bB^	72.71 ± 1.44 ^bcA^	76.85 ± 3.03 ^deB^	75.19 ± 1.65 ^cdA^	77.91 ± 2.56 ^eB^
7	59.32 ± 2.53 ^aA^	67.63 ± 2.32 ^bA^	70.66 ± 1.87 ^cA^	68.87 ± 1.87 ^bcA^	87.12 ± 0.47 ^dB^	67.98 ± 1.93 ^bA^
a*						
0	5.33 ± 0.97 ^cA^	6.90 ± 0.20 ^dA^	5.05 ± 0.46 ^cA^	1.28 ± 0.27 ^aA^	2.49 ± 0.20 ^bA^	1.22 ± 0.24 ^aA^
7	4.67 ± 0.30 ^dA^	6.44 ± 0.48 ^fA^	5.31 ± 0.35 ^eA^	1.74 ± 0.05 ^aB^	2.68 ± 0.22 ^cA^	2.15 ± 0.08 ^bB^
b*						
0	24.14 ± 0.62 ^abA^	27.93 ± 1.12 ^cA^	23.74 ± 0.77 ^aA^	23.40 ± 1.01 ^aB^	24.73 ± 0.31 ^bA^	24.36 ± 0.81 ^abA^
7	22.99 ± 0.27 ^bA^	28.09 ± 0.87 ^dA^	24.13 ± 0.82 ^cA^	21.19 ± 0.73 ^aA^	28.01 ± 0.27 ^dB^	23.74 ± 1.24 ^bcA^
pH						
0	7.67 ± 0.12 ^dA^	6.05 ± 0.09 ^aA^	6.74 ± 0.14 ^bA^	8.04 ± 0.10 ^eA^	6.12 ± 0.08 ^aA^	7.17 ± 0.12 ^cA^
7	7.65 ± 0.05 ^dA^	6.05 ± 0.06 ^aA^	6.83 ± 0.04 ^bA^	8.36 ± 0.08 ^eB^	6.07 ± 0.07 ^aA^	7.11 ± 0.06 ^cA^
Total fluid release (%)						
0	2.43 ± 0.18 ^dA^	10.47 ± 0.56 ^eA^	0.75 ± 0.04 ^bcA^	0.49 ± 0.03 ^abA^	1.14 ± 0.06 ^cA^	0.11 ± 0.01 ^aA^
7	11.89 ± 0.44 ^dB^	17.61 ± 0.56 ^eB^	2.83 ± 0.11 ^cB^	1.93 ± 0.09 ^bB^	3.14 ± 0.18 ^cB^	0.18 ± 0.01 ^aB^

* Different lowercase letters in the same row indicate significant differences between emulsion gel formulations (*p* < 0.05) for the same storage period, while different uppercase letters in the same column are indicative of significant differences between sampling times for the same emulsion gel formulation (*p* < 0.05); EG-PPI-CH, EG-PPI-P, EG-PPI-X—emulsion gels made with pea protein isolate and chitosan, pectin and xanthan, respectively; EG-SPI-CH, EG-SPI-P, EG-SPI-X—emulsion gels made with soy protein isolate and chitosan, pectin and xanthan, respectively.

**Table 2:** Texture parameters of emulsion gels at 0 and 7 days of storage at 4 °C *.

Storage Time (Days)	EG-PPI-CH	EG-PPI-P	EG-PPI-X	EG-SPI-CH	EG-SPI-P	EG-SPI-X
Hardness (N)						
0	22.96 ± 1.19 ^cB^	14.77 ± 0.51 ^aB^	16.66 ± 0.17 ^aB^	32.88 ± 1.45 ^dB^	18.53 ± 0.63 ^bB^	16.47 ± 0.95 ^aA^
7	17.76 ± 0.78 ^cA^	13.10 ± 0.74 ^aA^	15.21 ± 0.86 ^bA^	29.87 ± 1.07 ^dA^	15.47 ± 0.91 ^bA^	16.28 ± 0.84 ^bcA^
Cohesiveness (adm)						
0	0.94 ± 0.02 ^aB^	0.97 ± 0.01 ^aA^	0.93 ± 0.00 ^aA^	1.03 ± 0.01 ^aB^	0.99 ± 0.03 ^aA^	0.99 ± 0.01 ^aA^
7	0.76 ± 0.04 ^aA^	0.96 ± 0.05 ^bA^	1.00 ± 0.01 ^bcB^	0.93 ± 0.08 ^bA^	1.08 ± 0.08 ^cA^	1.02 ± 0.07 ^bcA^
Springiness (adm)						
0	0.94 ± 0.03 ^aA^	0.98 ± 0.00 ^bA^	0.99 ± 0.01 ^bA^	0.99 ± 0.04 ^bA^	0.98 ± 0.02 ^bA^	0.95 ± 0.01 ^abA^
7	1.00 ± 0.00 ^abB^	0.95 ± 0.05 ^aA^	1.04 ± 0.03 ^bB^	1.02 ± 0.00 ^bA^	0.99 ± 0.04 ^abA^	1.00 ± 0.05 ^abA^
Adhesiveness (J)						
0	−33.27 ± 1.21 ^fA^	−79.84 ± 0.69 ^cA^	−66.38 ± 1.59 ^dA^	−55.58 ± 1.73 ^eA^	−97.34 ± 1.72 ^aA^	−88.11 ± 0.17 ^bA^
7	−30.63 ± 1.77 ^fA^	−78.67 ± 1.77 ^cA^	−65.23 ± 2.61 ^dA^	−52.87 ± 1.49 ^eA^	−95.71 ± 2.28 ^aA^	−85.24 ± 2.10 ^bA^
Resilience (adm)						
0	22.34 ± 2.15 ^bcB^	19.24 ± 2.16 ^abA^	16.86 ± 2.83 ^aA^	24.59 ± 1.05 ^cA^	23.59 ± 0.74 ^cB^	21.57 ± 0.44 ^bcA^
7	14.33 ± 0.46 ^aA^	20.40 ± 1.15 ^bA^	19.36 ± 1.31 ^bA^	24.64 ± 1.63 ^cA^	20.12 ± 1.81 ^bA^	20.38 ± 0.90 ^bA^
Gumminess (N)						
0	21.66 ± 0.48 ^dB^	18.01 ± 0.77 ^cB^	14.12 ± 0.15 ^aA^	29.27 ± 1.68 ^eA^	14.60 ± 0.59 ^aA^	16.19 ± 0.76 ^bA^
7	13.51 ± 0.78 ^aA^	14.92 ± 0.43 ^bA^	17.14 ± 0.65 ^cB^	30.83 ± 0.76 ^dA^	14.14 ± 0.32 ^abA^	16.89 ± 0.90 ^cA^
Chewiness (N)						
0	20.25 ± 0.73 ^cB^	17.72 ± 0.80 ^bB^	14.02 ± 0.25 ^aA^	29.18 ± 2.32 ^dA^	14.36 ± 0.36 ^aA^	15.41 ± 0.87 ^aA^
7	13.51 ± 0.78 ^aA^	14.55 ± 0.78 ^aA^	17.85 ± 0.71 ^bB^	31.37 ± 0.96 ^cA^	14.05 ± 0.83 ^aA^	16.77 ± 0.56 ^bA^

* Different lowercase letters in the same row indicate significant differences between emulsion gel formulations (*p* < 0.05) for the same storage period, while different uppercase letters in the same column are indicative of significant differences between sampling times for the same emulsion gel formulation (*p* < 0.05): EG-PPI-CH, EG-PPI-P, EG-PPI-X—emulsion gels made with pea protein isolate and chitosan, pectin and xanthan, respectively; EG-SPI-CH, EG-SPI-P, EG-SPI-X—emulsion gels made with soy protein isolate and chitosan, pectin and xanthan, respectively.

**Table 3:** Proximate composition and energy values of control and reformulated burgers *.

	Control	PPI-CH	PPI-P	PPI-X	SPI-CH	SPI-P	SPI-X
Moisture (%)	53.30 ± 0.66 ^a^	56.85 ± 0.58 ^cd^	55.36 ± 0.77 ^b^	60.64 ± 0.65 ^e^	57.71 ± 0.75 ^d^	56.37 ± 0.67 ^bc^	61.58 ± 0.82 ^e^
Protein (%)	23.57 ± 0.33 ^b^	26.59 ± 0.39 ^d^	26.92 ± 0.41 ^d^	22.98 ± 0.38 ^b^	25.11 ± 0.42 ^c^	25.68 ± 0.34 ^c^	20.38 ± 0.27 ^a^
Fat (%)	17.22 ± 0.36 ^d^	10.46 ± 0.27 ^a^	13.52 ± 0.33 ^c^	11.29 ± 0.28 ^b^	11.27 ± 0.35 ^b^	13.26 ± 0.39 ^c^	11.70 ± 0.30 ^b^
Ash (%)	2.46 ± 0.12 ^c^	2.26 ± 0.13 ^ab^	2.15 ± 0.09 ^a^	2.70 ± 0.10 ^d^	2.24 ± 0.09 ^ab^	2.33 ± 0.07 ^bc^	2.51 ± 0.08 ^c^
Energy value (kcal/100 g)	267.48 ± 1.40 ^e^	219.95 ± 1.56 ^b^	241.81 ± 1.88 ^d^	206.76 ± 1.67 ^a^	220.56 ± 1.69 ^b^	235.63 ± 1.08 ^c^	205.77 ± 2.19 ^a^
Energy from fat (kcal/100 g)	156.70 ± 3.28 ^d^	95.19 ± 2.46 ^a^	123.03 ± 3.00 ^c^	102.74 ± 2.55 ^b^	102.56 ± 3.19 ^b^	120.67 ± 3.55 ^c^	106.47 ± 2.73 ^b^
Fat reduction (%)	-	39.26 ± 0.30 ^e^	21.49% ± 0.28 ^a^	34.44 ± 0.26 ^d^	34.56 ± 0.66 ^d^	23.01 ± 0.56 ^b^	32.06 ± 0.32 ^c^
Energy value reduction (%)	-	17.77 ± 0.15 ^c^	9.60 ± 0.23 ^a^	22.70 ± 0.22 ^d^	17.55 ± 0.20 ^c^	11.91 ± 0.06 ^b^	23.07 ± 0.42 ^d^

* Different lowercase letters in the same row indicate significant differences between formulations (*p* < 0.05); Control—control burgers; PPI-CH, PPI-P and PPI-X—burgers reformulated with EG-PPI-CH, EG-PPI-P and EG-PPI-X, respectively; SPI-CH, SPI-P and SPI-X—burgers reformulated with EG-SPI-CH, EG-SPI-P and EG-SPI-X, respectively.

**Table 4:** Fatty acid profile (expressed as g/100 g of total fatty acids) and nutritional indices of control and reformulated burgers *.

Fatty Acids	Control	PPI-CH	PPI-P	PPI-X	SPI-CH	SPI-P	SPI-X
Butyric (C4:0)	2.21 ± 0.06 ^b^	0.00 ± 0.00 ^a^	0.00 ± 0.00 ^a^	0.00 ± 0.00 ^a^	0.00 ± 0.00 ^a^	0.00 ± 0.00 ^a^	0.01 ± 0.01 ^a^
Caproic (C6:0)	2.14 ± 0.08 ^d^	0.45 ± 0.02 ^a^	0.55 ± 0.03 ^bc^	0.58 ± 0.02 ^c^	0.46 ± 0.01 ^a^	0.54 ± 0.02 ^bc^	0.51 ± 0.02 ^ab^
Caprylic (C8:0)	0.92 ± 0.04 ^d^	0.46 ± 0.02 ^b^	0.49 ± 0.02 ^bc^	0.50 ± 0.03 ^bc^	0.40 ± 0.01 ^a^	0.52 ± 0.02 ^c^	0.48 ± 0.02 ^bc^
Capric (C10:0)	0.63 ± 0.03 ^d^	0.26 ± 0.01 ^ab^	0.29 ± 0.02 ^b^	0.33 ± 0.02 ^c^	0.25 ± 0.01 ^a^	0.26 ± 0.01 ^ab^	0.28 ± 0.02 ^ab^
Lauric (C12:0)	0.22 ± 0.02 ^c^	0.04 ± 0.01 ^a^	0.05 ± 0.01 ^ab^	0.05 ± 0.01 ^ab^	0.05 ± 0.01 ^ab^	0.07 ± 0.01 ^b^	0.07 ± 0.01 ^b^
Myristic (C14:0)	2.55 ± 0.11 ^d^	0.53 ± 0.03 ^c^	0.51 ± 0.02 ^bc^	0.47 ± 0.02 ^bc^	0.35 ± 0.02 ^a^	0.36 ± 0.02 ^a^	0.43 ± 0.02 ^ab^
Myristoleic (C14:1)	0.12 ± 0.02 ^e^	0.05 ± 0.01 ^bc^	0.04 ± 0.01 ^ab^	0.05 ± 0.01 ^bc^	0.02 ± 0.01 ^a^	0.07 ± 0.01 ^cd^	0.08 ± 0.01 ^d^
Pentadecanoic (C15:0)	0.72 ± 0.05 ^c^	0.16 ± 0.01 ^a^	0.17 ± 0.01 ^ab^	0.21 ± 0.02 ^b^	0.16 ± 0.01 ^a^	0.16 ± 0.02 ^a^	0.18 ± 0.02 ^ab^
Pentadecenoic (C15:1n-13)	1.32 ± 0.07 ^d^	0.36 ± 0.02 ^abc^	0.38 ± 0.03 ^bc^	0.30 ± 0.02 ^a^	0.38 ± 0.02 ^bc^	0.33 ± 0.02 ^ab^	0.41 ± 0.03 ^c^
Palmitic (C16:0)	27. 95 ± 1.22 ^b^	11.91 ± 0.58 ^a^	11.76 ± 0.46 ^a^	11.85 ± 0.56 ^a^	11.80 ± 0.70 ^a^	11.75 ± 0.43 ^a^	11.53 ± 0.51 ^a^
Palmitoleic (C16:1n-7)	2.25 ± 0.1 ^d^	1.26 ± 0.07 ^c^	1.09 ± 0.05 ^ab^	1.03 ± 0.06 ^a^	1.19 ± 0.07 ^bc^	1.19 ± 0.05 ^bc^	1.27 ± 0.08 ^c^
Heptadecanoic (C17:0)	0.74 ± 0.03 ^c^	0.20 ± 0.04 ^b^	0.17 ± 0.01 ^ab^	0.20 ± 0.02 ^b^	0.15 ± 0.01 ^a^	0.15 ± 0.01 ^a^	0.17 ± 0.02 ^ab^
Heptadecenoic (C17:1n-7)	0.36 ± 0.03 ^d^	0. 25 ± 0.02 ^ab^	0.28 ± 0.02 ^bc^	0.31 ± 0.02 ^c^	0.25 ± 0.02 ^ab^	0.22 ± 0.02 ^a^	0.24 ± 0.02 ^a^
Stearic (C18:0)	13.19 ± 0.47 ^b^	4.73 ± 0.21 ^a^	4.64 ± 0.24 ^a^	4.80 ± 0.25 ^a^	4.68 ± 0.19 ^a^	4.73 ± 0.16 ^a^	4.76 ± 0.22 ^a^
Oleic (C18:1n-9)	35.18 ± 1.18 ^a^	47.62 ± 1.59 ^b^	47.40 ± 1.26 ^b^	47.28 ± 1.46 ^b^	47.57 ± 1.37 ^b^	46.85 ± 1.20 ^b^	47.33 ± 1.35 ^b^
Linoleic (C18:2n-6)	5.17 ± 0.26 ^a^	10.17 ± 0.44 ^b^	10.22 ± 0.38 ^b^	10.16 ± 0.28 ^b^	10.36 ± 0.34 ^b^	10.50 ± 0.33 ^b^	10.45 ± 0.34 ^b^
γ Linolenic (C18:3n-6)	0.00 ± 0.00 ^a^	0.08 ± 0.01 ^b^	0.11 ± 0.01 ^c^	0.13 ± 0.02 ^d^	0.09 ± 0.01 ^b^	0.12 ± 0.01 ^cd^	0.12 ± 0.01 ^cd^
α Linolenic (C18:3n-3)	0.13 ± 0.02 ^a^	17.31 ± 0.47 ^b^	17.45 ± 0.56 ^b^	17.32 ± 0.52 ^b^	17.40 ± 0.38 ^b^	17.55 ± 0.67 ^b^	17.40 ± 0.63 ^b^
Conjugated linoleic acid (CLA) (C18:3n-3)	0.10 ± 0.01 ^a^	0.24 ± 0.02 ^c^	0.21 ± 0.02 ^bc^	0.19 ± 0.02 ^b^	0.19 ± 0.01 ^b^	0.24 ± 0.02 ^c^	0.20 ± 0.02 ^b^
Octadecatetraenoic (C18:4n-3)	1.18 ± 0.08 ^d^	0.26 ± 0.02 ^abc^	0.24 ± 0.02 ^ab^	0.21 ± 0.02 ^a^	0.31 ± 0.03 ^c^	0.29 ± 0.03 ^bc^	0.26 ± 0.02 ^abc^
Arachic (C20:0)	0.03 ± 0.01 ^a^	0.10 ± 0.01 ^bc^	0.12 ± 0.02 ^c^	0.08 ± 0.01 ^b^	0.10 ± 0.01 ^bc^	0.10 ± 0.01 ^bc^	0.09 ± 0.01 ^b^
Eicosadienoic (C20:2n-6)	1.75 ± 0.08 ^b^	0.06 ± 0.01 ^a^	0.05 ± 0.01 ^a^	0.06 ± 0.01 ^a^	0.06 ± 0.01 ^a^	0.08 ± 0.02 ^a^	0.06 ± 0.01 ^a^
Dihomo-γ-linolenic (C20:3n-6)	0.13 ± 0.01 ^ab^	0.12 ± 0.01 ^a^	0.12 ± 0.01 ^a^	0.17 ± 0.02 ^c^	0.14 ± 0.02 ^ab^	0.17 ± 0.02 ^c^	0.15 ± 0.01 ^bc^
Eicosatrienoic (Mead) (C20:3n-3)	0.00 ± 0.00 ^a^	0.14 ± 0.02 ^b^	0.15 ± 0.02 ^b^	0.14 ± 0.01 ^b^	0.18 ± 0.02 ^c^	0.14 ± 0.01 ^b^	0.18 ± 0.02 ^c^
Arachidonic (C20:4n-6)	0.05 ± 0.01 ^a^	0.34 ± 0.03 ^b^	0.39 ± 0.03 ^b^	0.37 ± 0.04 ^b^	0.37 ± 0.03 ^b^	0.39 ± 0.03 ^b^	0.37 ± 0.03 ^b^
Tricosanoic (C23:0)	0.05 ± 0.01 ^b^	0.00 ± 0.00 ^a^	0.00 ± 0.00 ^a^	0.00 ± 0.00 ^a^	0.00 ± 0.00 ^a^	0.00 ± 0.00 ^a^	0.00 ± 0.00 ^a^
Docosadienoic (C22:2n-6)	0.00 ± 0.00 ^a^	0.12 ± 0.02 ^b^	0.15 ± 0.02 ^bc^	0.13 ± 0.01 ^bc^	0.16 ± 0.02 ^c^	0.16 ± 0.03 ^c^	0.15 ± 0.02 ^bc^
Docosatrienoic (C22:3n-6)	0.00 ± 0.00 ^a^	0.00 ± 0.00 ^a^	0.00 ± 0.00 ^a^	0.00 ± 0.00 ^a^	0.00 ± 0.00 ^a^	0.00 ± 0.00 ^a^	0.00 ± 0.00 ^a^
Eicosapentaenoic (C22:5n-3)	0.10 ± 0.01 ^a^	0.10 ± 0.01 ^a^	0.14 ± 0.02 ^b^	0.14 ± 0.02 ^b^	0.10 ± 0.02 ^a^	0.13 ± 0.02 ^ab^	0.12 ± 0.02 ^ab^
Lignoceric (C24:0)	0.10 ± 0.01 ^a^	0.16 ± 0.02 ^bc^	0.17 ± 0.02 ^c^	0.16 ± 0.02 ^bc^	0.13 ± 0.02 ^ab^	0.13 ± 0.02 ^ab^	0.14 ± 0.02 ^bc^
Nervonic (C24:1n-9)	0.00 ± 0.00 ^a^	1.78 ± 0.09 ^b^	1.80 ± 0.11 ^b^	1.89 ± 0.08 ^b^	1.85 ± 0.12 ^b^	1.92 ± 0.14 ^b^	1.75 ± 0.10 ^b^
Docosatetraenoic (C22:4n-6)	0.00 ± 0.00 ^a^	0.00 ± 0.00 ^a^	0.00 ± 0.00 ^a^	0.00 ± 0.00 ^a^	0.00 ± 0.00 ^a^	0.00 ± 0.00 ^a^	0.00 ± 0.00 ^a^
Clupanodonic (C22:5n-3)	0.00 ± 0.00 ^a^	0.12 ± 0.02 ^b^	0.11 ± 0.03 ^b^	0.12 ± 0.02 ^b^	0.14 ± 0.03 ^b^	0.13 ± 0.02 ^b^	0.14 ± 0.02 ^b^
Docosahexaenoic (C22:6n-3)	0.00 ± 0.00 ^a^	0.06 ± 0.01 ^b^	0.06 ± 0.01 ^b^	0.07 ± 0.01 ^b^	0.06 ± 0.01 ^b^	0.06 ± 0.01 ^b^	0.07 ± 0.01 ^b^
Other fatty acids	0.71 ± 0.05 ^c^	0.56 ± 0.04 ^a^	0.69 ± 0.05 ^bc^	0.70 ± 0.06 ^c^	0.65 ± 0.07 ^abc^	0.68 ± 0.04 ^bc^	0.60 ± 0.05 ^ab^
Nutritional indices							
	Control	PPI-CH	PPI-P	PPI-X	SPI-CH	SPI-P	SPI-X
Σ SFA	51.45 ± 2.11 ^b^	19.00 ± 0.92 ^a^	18.92 ± 0.82 ^a^	19.23 ± 0.94 ^a^	18.53 ± 0.96 ^a^	18.77 ± 0.69 ^a^	18.65 ± 0.85 ^a^
Σ MUFA	39.23 ± 1.40 ^a^	51.32 ± 1.80 ^b^	50.99 ± 1.48 ^b^	50.86 ± 1.65 ^b^	51.26 ± 1.61 ^b^	50.58 ± 1.44 ^b^	51.08 ± 1.59 ^b^
Σ PUFA	8.61 ± 0.48 ^a^	29.12 ± 1.05 ^b^	29.40 ± 1.10 ^b^	29.21 ± 0.98 ^b^	29.56 ± 0.89 ^b^	29.96 ± 1.20 ^b^	29.67 ± 1.12 ^b^
Σ PUFA n-6	7.20 ± 0.37 ^b^	11.13 ± 0.53 ^b^	11.25 ± 0.47 ^b^	11.21 ± 0.38 ^b^	11.37 ± 0.43 ^b^	11.66 ± 0.45 ^b^	11.50 ± 0.43 ^b^
Σ PUFA n-3	1.41 ± 0.11 ^a^	17.99 ± 0.51 ^b^	18.15 ± 0.62 ^b^	18.00 ± 0.58 ^b^	18.19 ± 0.45 ^b^	18.30 ± 0.74 ^b^	18.17 ± 0.68 ^b^
n-6/n-3	5.11 ^b^	0.62 ^a^	0.62 ^a^	0.62 ^a^	0.62 ^a^	0.64 ^a^	0.63 ^a^
AI	0.80 ^b^	0.17 ^a^	0.17 ^a^	0.17 ^a^	0.16 ^a^	0.16 ^a^	0.16 ^a^
TI	1.34 ^b^	0.20 ^a^	0.20 ^a^	0.20 ^a^	0.19 ^a^	0.19 ^a^	0.19 ^a^
h/H	1.33 ^a^	6.09 ^b^	6.17 ^b^	6.12 ^b^	6.26 ^b^	6.24 ^b^	6.35 ^b^

* Different lowercase letters in the same row indicate significant differences between formulations (*p* < 0.05); Control—control burgers; PPI-CH, PPI-P and PPI-X—burgers reformulated with EG-PPI-CH, EG-PPI-P and EG-PPI-X, respectively; SPI-CH, SPI-P and SPI-X—burgers reformulated with EG-SPI-CH, EG-SPI-P and EG-SPI-X, respectively.

**Table 5:** Texture parameters of control and reformulated burgers at 0 and 10 days of storage at 4 °C*.

Storage Time (Days)	Control	PPI-CH	PPI-P	PPI-X	SPI-CH	SPI-P	SPI-X
Hardness (N)							
0	21.36 ± 1.02 ^cA^	73.03 ± 3.40 ^fA^	48.36 ± 1.36 ^dA^	11.01 ± 0.76 ^bB^	63.39 ± 3.46 ^eA^	45.67 ± 1.68 ^dA^	6.99 ± 0.48 ^aB^
10	24.10 ± 1.24 ^bB^	67.30 ± 3.24 ^fA^	48.52 ± 1.40 ^dA^	4.96 ± 1.65 ^aA^	63.98 ± 1.50 ^eA^	42.67 ± 1.29 ^cA^	5.08 ± 0.22 ^aA^
Cohesiveness (adm)							
0	0.13 ± 0.02 ^aA^	0.60 ± 0.02 ^eB^	0.31 ± 0.02 ^bA^	0.36 ± 0.04 ^cB^	0.53 ± 0.02 ^dB^	0.29 ± 0.02 ^bA^	0.16 ± 0.01 ^aA^
10	0.22 ± 0.05 ^aB^	0.41 ± 0.11 ^bA^	0.41 ± 0.05 ^bB^	0.13 ± 0.08 ^aA^	0.47 ± 0.08 ^bA^	0.41 ± 0.09 ^bB^	0.15 ± 0.03 ^aA^
Springiness (adm)							
0	1.00 ± 0.00 ^aA^	1.00 ± 0.00 ^aA^	1.00 ± 0.00 ^aA^	1.00 ± 0.00 ^aA^	1.00 ± 0.00 ^aA^	1.00 ± 0.00 ^aA^	1.00 ± 0.00 ^aA^
10	1.00 ± 0.00 ^aA^	1.00 ± 0.00 ^aA^	1.00 ± 0.00 ^aA^	1.00 ± 0.00 ^aA^	1.00 ± 0.00 ^aA^	1.00 ± 0.00 ^aA^	1.00 ± 0.00 ^aA^
Adhesiveness (J)							
0	−52.85 ± 2.90 ^aA^	−12.88 ± 0.36 ^eB^	−23.85 ± 1.51 ^dA^	−7.10 ± 0.56 ^fA^	−40.10 ± 2.30 ^cA^	−43.35 ± 2.31 ^bA^	−14.14 ± 1.09 ^eA^
10	−25.33 ± 0.74 ^bB^	−27.45 ± 1.00 ^aA^	−20.22 ± 0.55 ^cB^	−1.57 ± 0.11 ^eB^	−20.67 ± 1.26 ^cB^	−16.43 ± 1.13 ^dB^	−1.74 ± 0.54 ^eB^
Resilience (adm)							
0	0.13 ± 0.01 ^aA^	0.44 ± 0.02 ^cB^	0.74 ± 0.04 ^fB^	0.18 ± 0.01 ^bA^	0.59 ± 0.04 ^eB^	0.53 ± 0.02 ^dB^	0.25 ± 0.03 ^bB^
10	0.23 ± 0.02 ^bcB^	0.29 ± 0.04 ^cA^	0.23 ± 0.04 ^bcA^	0.17 ± 0.02 ^bA^	0.21 ± 0.04 ^bA^	0.11 ± 0.01 ^aA^	0.17 ± 0.05 ^bA^
Gumminess (N)							
0	2.28 ± 0.22 ^abA^	43.96 ± 1.86 ^fB^	15.49 ± 0.59 ^dA^	3.11 ± 0.40 ^bB^	33.01 ± 0.13 ^eB^	13.12 ± 0.53 ^cA^	1.20 ± 0.17 ^aB^
10	5.19 ± 0.48 ^aB^	27.31 ± 1.55 ^cA^	19.74 ± 1.31 ^bB^	0.66 ± 0.37 ^aA^	30.28 ± 1.68 ^cA^	17.55 ± 2.58 ^bB^	0.77 ± 0.09 ^aA^
Chewiness (N)							
0	2.28 ± 0.23 ^abA^	44.02 ± 1.91 ^fB^	15.50 ± 0.57 ^dA^	3.11 ± 0.41 ^bB^	33.07 ± 0.13 ^eB^	13.14 ± 0.51 ^cA^	1.21 ± 0.17 ^aB^
10	5.20 ± 0.49 ^aB^	27.34 ± 1.53 ^cA^	19.75 ± 1.33 ^bB^	0.79 ± 0.14 ^aA^	30.32 ± 1.70 ^cA^	17.54 ± 2.56 ^bB^	0.77 ± 0.09 ^aA^
Shear force (N)							
0	46.81 ± 1.02 ^bB^	78.62 ± 0.45 ^dB^	77.26 ± 0.74 ^cB^	22.58 ± 0.50 ^aA^	97.83 ± 0.58 ^fB^	87.24 ± 0.77 ^eB^	21.84 ± 0.49 ^aB^
10	38.82 ± 1.83 ^bA^	65.36 ± 2.02 ^dA^	58.82 ± 2.94 ^cA^	21.57 ± 0.90 ^aA^	86.87 ± 2.84 ^fA^	80.74 ± 1.87 ^eA^	19.19 ± 0.63 ^aA^

* Different lowercase letters in the same row indicate significant differences between burger formulations (*p* < 0.05) for the same storage period, while different uppercase letters in the same column are indicative of significant differences between sampling times for the same burger formulation (*p* < 0.05); Control—control burgers; PPI-CH, PPI-P and PPI-X—burgers reformulated with EG-PPI-CH, EG-PPI-P and EG-PPI-X, respectively; SPI-CH, SPI-P and SPI-X—burgers reformulated with EG-SPI-CH, EG-SPI-P and EG-SPI-X, respectively.

**Table 6:** Color parameters (L*—lightness, a*—redness and b*—yellowness) and pH of control and reformulated burgers at 0, 5 and 10 days of storage at 4 °C *.

Storage Time (Days)	Control	PPI-CH	PPI-P	PPI-X	SPI-CH	SPI-P	SPI-X
L*							
0	53.88 ± 2.66 ^cC^	45.93 ± 1.81 ^abB^	44.20 ± 2.64 ^aA^	53.03 ± 1.61 ^cB^	47.62 ± 1.04 ^bB^	46.40 ± 0.94 ^bA^	52.12 ± 0.89 ^cB^
5	47.95 ± 2.58 ^bcB^	39.38 ± 2.28 ^aA^	46.04 ± 2.17 ^bAB^	49.70 ± 1.62 ^cdA^	49.87 ± 1.80 ^cdC^	49.13 ± 0.48 ^cdB^	50.38 ± 0.45 ^dA^
10	43.85 ± 1.63 ^aA^	48.33 ± 1.67 ^bcC^	47.85 ± 1.95 ^bB^	50.61 ± 0.83 ^dA^	44.04 ± 0.98 ^aA^	46.81 ± 1.08 ^bA^	49.59 ± 0.85 ^cdA^
a*							
0	9.54 ± 0.82 ^bB^	10.74 ± 0.41 ^cC^	9.36 ± 0.44 ^abC^	9.07 ± 0.31 ^abC^	10.59 ± 0.27 ^cC^	9.10 ± 0.29 ^abB^	8.91 ± 0.25 ^aC^
5	6.02 ± 0.64 ^aA^	10.18 ± 0.44 ^eB^	8.53 ± 0.36 ^cdB^	8.16 ± 0.29 ^cB^	9.74 ± 0.31 ^eB^	8.84 ± 0.26 ^dB^	7.68 ± 0.25 ^bB^
10	5.35 ± 0.53 ^aA^	9.55 ± 0.31 ^eA^	7.74 ± 0.41 ^cA^	6.98 ± 0.12 ^bA^	8.44 ± 0.21 ^dA^	8.09 ± 0.15 ^cdA^	7.12 ± 0.40 ^bA^
b*							
0	16.35 ± 1.10 ^aB^	18.20 ± 1.06 ^cA^	17.72 ± 0.43 ^bcA^	15.96 ± 0.50 ^aA^	18.03 ± 0.05 ^cA^	17.14 ± 0.23 ^bA^	16.14 ± 0.54 ^aA^
5	15.09 ± 0.57 ^aA^	20.06 ± 1.30 ^dB^	18.90 ± 0.21 ^cB^	16.34 ± 0.60 ^bA^	20.34 ± 0.43 ^dC^	18.19 ± 0.50 ^cB^	16.30 ± 0.30 ^bA^
10	15.20 ± 0.58 ^aA^	20.69 ± 0.21 ^eB^	19.25 ± 1.42 ^dB^	17.03 ± 0.40 ^bB^	19.79 ± 0.24 ^dB^	18.44 ± 0.22 ^cB^	17.16 ± 0.60 ^bB^
pH							
0	6.11 ± 0.05 ^aA^	6.49 ± 0.08 ^bA^	6.04 ± 0.04 ^aA^	6.13 ± 0.05 ^aA^	6.40 ± 0.07 ^bA^	6.03 ± 0.07 ^aA^	6.11 ± 0.06 ^aA^
5	6.12 ± 0.06 ^aA^	6.58 ± 0.08 ^bA^	6.15 ± 0.08 ^aB^	6.18 ± 0.05 ^aA^	6.48 ± 0.07 ^bA^	6.07 ± 0.04 ^aA^	6.13 ± 0.06 ^aA^
10	6.12 ± 0.04 ^abA^	6.54 ± 0.06 ^dA^	6.16 ± 0.03 ^abcB^	6.21 ± 0.04 ^cA^	6.52 ± 0.04 ^dA^	6.09 ± 0.03 ^aA^	6.18 ± 0.04 ^bcA^

* Different lowercase letters in the same row indicate significant differences between burger formulations (*p* < 0.05) for the same storage period, while different uppercase letters in the same column are indicative of significant differences between sampling times for the same burger formulation (*p* < 0.05); Control—control burgers; PPI-CH, PPI-P and PPI-X—burgers reformulated with EG-PPI-CH, EG-PPI-P and EG-PPI-X, respectively; SPI-CH, SPI-P and SPI-X—burgers reformulated with EG-SPI-CH, EG-SPI-P and EG-SPI-X, respectively.

**Table 7:** Technological properties of control and reformulated burgers *.

	Control	PPI-CH	PPI-P	PPI-X	SPI-CH	SPI-P	SPI-X
Cooking loss (%)	38.96 ± 0.71 ^d^	36.49 ± 0.61 ^c^	39.23 ± 1.19 ^d^	26.57 ± 2.54 ^b^	36.51 ± 1.00 ^c^	39.57 ± 1.67 ^d^	25.24 ± 1.06 ^a^
Shrinkage (%)	23.64 ± 0.93 ^e^	20.83 ± 0.55 ^c^	22.24 ± 0.85 ^d^	13.45 ± 0.47 ^b^	22.83 ± 0.78 ^de^	20.79 ± 0.67 ^c^	9.69 ± 0.39 ^a^
Moisture retention (%)	32.53 ± 0.40 ^a^	36.11 ± 0.37 ^c^	33.64 ± 0.47 ^b^	44.53 ± 0.48 ^d^	36.64 ± 0.48 ^c^	34.06 ± 0.40 ^b^	45.04 ± 0.61 ^d^
Fat retention (%)	75.55 ± 1.37 ^a^	91.76 ± 1.51 ^bc^	91.40 ± 1.15 ^b^	94.22 ± 1.44 ^cd^	90.37 ± 1.76 ^b^	91.78 ± 1.78 ^bc^	94.83 ± 1.64 ^d^

* Different lowercase letters in the same row indicate significant differences between formulations (*p* < 0.05); Control—control burgers; PPI-CH, PPI-P and PPI-X—burgers reformulated with EG-PPI-CH, EG-PPI-P and EG-PPI-X, respectively; SPI-CH, SPI-P and SPI-X—burgers reformulated with EG-SPI-CH, EG-SPI-P and EG-SPI-X, respectively.

**Table 8:** Sensory attributes and overall acceptability scores of control and reformulated burgers *.

	Control	PPI-CH	PPI-P	PPI-X	SPI-CH	SPI-P	SPI-X
Appearance	7.75 ± 0.62 ^bc^	7.92 ± 0.51 ^c^	8.00 ± 0.74 ^c^	7.25 ± 0.45 ^a^	7.58 ± 0.51 ^abc^	7.92 ± 0.67 ^c^	7.33 ± 0.49 ^ab^
Taste	8.00 ± 0.60 ^a^	7.67 ± 0.49 ^a^	7.75 ± 0.45 ^a^	7.83 ± 0.58 ^a^	7.67 ± 0.65 ^a^	7.92 ± 0.51 ^a^	7.83 ± 0.83 ^a^
Flavor	7.83 ± 0.72 ^a^	7.50 ± 0.52 ^a^	7.42 ± 0.67 ^a^	7.58 ± 0.51 ^a^	7.33 ± 0.65 ^a^	7.33 ± 0.65 ^a^	7.67 ± 0.89 ^a^
Texture	7.83 ± 0.72 ^b^	7.92 ± 0.51 ^b^	7.42 ± 0.51 ^b^	6.25 ± 0.75 ^a^	7.92 ± 0.67 ^b^	7.58 ± 0.67 ^b^	6.33 ± 0.65 ^a^
General acceptability	8.08 ± 0.67 ^d^	7.92 ± 0.51 ^cd^	7.50 ± 0.52 ^bc^	6.67 ± 0.65 ^a^	7.83 ± 0.58 ^bcd^	7.42 ± 0.51 ^b^	6.50 ± 0.52 ^a^

* Different lowercase letters in the same row indicate significant differences between formulations (*p* < 0.05); Control—control burgers; PPI-CH, PPI-P and PPI-X—burgers reformulated with EG-PPI-CH, EG-PPI-P and EG-PPI-X, respectively; SPI-CH, SPI-P and SPI-X—burgers reformulated with EG-SPI-CH, EG-SPI-P and EG-SPI-X, respectively.

**Table 9:** Formulation (g/100 g) of different emulsion gels.

Formulation	EG-PPI-CH	EG-PPI-P	EG-PPI-X	EG-SPI-CH	EG-SPI-P	EG-SPI-X
Pea protein isolate (PPI)	16	16	16	-	-	-
Soy protein isolate (SPI)	-	-	-	10	10	10
Transglutaminase	1	1	1	1	1	1
Chitosan (CH)	3	-	-	3	-	-
Pectin (P)	-	3	-	-	3	-
Xanthan (X)	-	-	1	-	-	1
Water	40	40	42	46	46	48
Oil mixture	40	40	40	40	40	40

## Data Availability

Data are contained within the article.
